# Increasing Magnetic Anisotropy in Bimetallic Nanoislands Grown on fcc(111) Metal Surfaces

**DOI:** 10.3390/nano12030518

**Published:** 2022-02-02

**Authors:** Sergio Vlaic, Dimitris Mousadakos, Safia Ouazi, Stefano Rusponi, Harald Brune

**Affiliations:** 1 Institute of Physics, Ecole Polytechnique Fédérale de Lausanne, CH-1015 Lausanne, Switzerland; sergio.vlaic@espci.fr (S.V.); dimitris.mousadakos@epfl.ch (D.M.); ouazi.safia@gmail.com (S.O.); stefano.rusponi@epfl.ch (S.R.); 2 Laboratoire de Physique et d’Étude des Matériaux (LPEM), ESPCI Paris-PSL Universtity, CNRS UMR8213, Sorbonne Université, 75005 Paris, France

**Keywords:** blocking temperature, nanoislands, transition metals, magnetic susceptibility, magneto-optical Kerr effect, scanning tunneling microscopy

## Abstract

The magnetic properties and the atomic scale morphology of bimetallic two-dimensional nanoislands, epitaxially grown on fcc(111) metal surfaces, have been studied by means of Magneto-Optical Kerr Effect and Scanning Tunneling Microscopy. We investigate the effect on blocking temperature of one-dimensional interlines appearing in core-shell structures, of two-dimensional interfaces created by capping, and of random alloying. The islands are grown on Pt(111) and contain a Co-core, surrounded by Ag, Rh, and Pd shells, or capped by Pd. The largest effect is obtained by Pd capping, increasing the blocking temperature by a factor of three compared to pure Co islands. In addition, for Co-core Fe-shell and Co-core Fe_x_Co_1−x_-shell islands, self-assembled into well ordered superlattices on Au(11,12,12) vicinal surfaces, we find a strong enhancement of the blocking temperature compared to pure Co islands of the same size. These ultra-high-density (15 Tdots/in^2^) superlattices of CoFe nanodots, only 500 atoms in size, have blocking temperature exceeding 100 K. Our findings open new possibilities to tailor the magnetic properties of nanoislands.

## 1. Introduction

The increasing quantity of data generated requires a concomitant increase in storage capacity. While charge-based flash memories have replaced hard disk drives (HDD) in personal computers, magnetic information storage remains the primary choice for large quantities of data. In servers, HDDs are used due to the required fast access times, while, for long-term archives, e.g., in banks or insurance companies, magnetic tapes are chosen as the most economic and durable recording medium. In present magnetic recording media, every bit consists of many grains, each having a single magnetic domain and being a few nanometers in diameter. One of the main challenges in magnetic information storage is to increase the bit density without running into the superparamagnetic limit, where the magnetization reverses by thermal excitation [1,2]. This forces one to chose materials with high magnetic anisotropy energy (MAE) per atom, which requires large switching fields. Novel magnetic recording materials [3,4], bit patterned media (BPM) [5], and novel writing technologies, such as heat-assisted magnetic recording (HAMR) [4,6], are investigated to enable HDDs with increased storage densities.

Exploring the ultimate density limits of magnetic information storage requires a fundamental understanding of the magnetic properties of the constituent atoms of the grains or bits as a function of their coordination number and chemistry. As feature sizes get smaller, more and more atoms are located at interfaces between two chemically different regions of the storage material. Mostly, these interfaces are two-dimensional (2D); however, for sub-10 nm structures, a significant fraction of the atoms are located at one-dimensional (1D) interlines, i.e., at the edges of the nanostructures. The magnetic and electronic properties of atoms located at these low symmetry regions are so different from the ones in bulk that they may dominate the properties of the entire nanostructure [7,8].

This motivates the investigation of model systems, where the magnetic properties can be investigated as a function of the number of atoms located at different types of interfaces and interlines. A fruitful approach in this respect has been the epitaxial growth of 2D magnetic nanostructures at single crystal surfaces. By kinetically controlled growth, one can adjust their density, size, morphology, and composition [9]. The numbers of atoms located at the surface, interfaces, or interlines between different chemical elements can be determined with scanning tunneling microscopy (STM) and correlated with the magnetic properties, either assessed for each individual nanostructure by means of STM using spin-polarized tips [10], or investigated with spatially integrating techniques that measure the properties of the ensemble of nanostructures. In both ways, an atomic scale structure–magnetic property relationship can be established.

In this paper, we use the second approach. We determine the atomic structure of 2D magnetic nanostructures with STM and investigate, in-situ, the temperature dependent magnetic susceptibility χ(T) of the ensemble of nanostructures with magneto-optical Kerr effect (MOKE). We investigate mono- and bi-layer islands, smaller than 3000 atoms in size and grown on fcc(111) single-crystal surfaces, that induce an uniaxial out-of-plane magnetic easy axis anisotropy, minimizing mutual dipolar interactions. The atoms in each island are ferromagnetically ordered; therefore, the islands are single domain. Depending on size and shape, they either reverse their magnetization by coherent rotation of the magnetic moments of all constituent atoms, or by domain wall creation and motion. The blocking temperature $T_{b}$ marks the transition from stable magnetization to superparamagnetism. With the aim to increase $T_{b}$ or a given island size, we investigate lateral and vertical decoration of the islands with different elements. From comparison of the measured χ(T) curves with simulated ones that take the atomic structure of the ensemble as input, we identify the anisotropy energies of the differently coordinated constituent atoms. We determine which element combinations have largest 1D interline, and which largest 2D interface anisotropies, and show how this knowledge can be used to create the smallest nanostructures with stable magnetization.

The manuscript is divided in two parts. In the first, we investigate atomically sharp 2D interfaces and 1D interlines between 3*d* and 4*d* transition metals since 4*d* elements may significantly enhance the MAE of 3*d* elements. This is based on two arguments: (i) 4*d* elements are better suited than 5*d* elements since their *d*-band is more narrow, explaining why Ru ([Kr] $4d^{7}5s^{1}$) and Rh ([Kr] $4d^{8}5s^{1}$) are closer to the onset of magnetism than the corresponding 5*d* transition metals Pt ([Xe] $4f^{14}5d^{9}6s^{1}$) and Ir ([Xe] $4f^{14}5d^{7}6s^{2}$). This implies that large magnetic moments of up to $m=1\;\mu_{B}$ can be induced in 4*d* transition metals [11,12,13]. (ii) The spin-orbit coupling (SOC) of 4*d* elements is more than twice the one of 3*d* elements [14]; therefore, once they get spin-polarized, they are expected to strongly contribute to the MAE [11,15]. In bimetallic 3*d*–5*d* thin films, where large anisotropy manifests itself due to strong SOC at the interface [16], an additional 3*d*–4*d* interface is expected to increase the MAE even further. Motivated by these observations, we grew Co islands on Pt(111) and decorated them laterally, or capped them, with 4*d* transition metals. We selected Rh ([Kr] $4d^{8}5s^{1}$), Pd ([Kr] $4d^{10}$), and Ag ([Kr] $4d^{10}5s^{1}$), which are three adjacent elements in the same row of the periodic table. These electronic configurations induce different degrees of magnetic polarization, when the respective element hybridizes with a ferromagnetic material. Rh and Pd are considered as highly polarizable [17,18]; therefore, their hybridization at the interface or interline with the Co core should result in large, induced magnetic moment and contribute to the MAE of the nanoisland [7]. On the other hand, Ag usually shows very small polarization at the interface with a ferromagnetic element [19,20,21,22]. Hence, it is interesting to investigate whether an Ag interline (or interface) contributes to the MAE, or whether it simply acts as a passivating and protecting shell for the Co-core. Such a passivating layer is attractive when used in combination with a third element producing an MAE enhancement, e.g., at the interline and an MAE reduction at the interface. In this situation, the passivating element can be used to suppress the MAE reduction in order to only benefit from the MAE enhancement.

In the second part, we demonstrate the combination of spatial order of the magnetic nanostructures with their enhanced magnetic anisotropy. Spatial order is achieved by self-assembly of Co island superlattices on Au(111) vicinal surfaces used as templates [23,24,25]. These Co-core islands are then decorated by Fe shells to benefit from the interline anisotropy between the two elements [26], and, for comparison, we also create Co-core CoFe alloy shell islands, all on Au(11,12,12). Compared to pure Co islands of identical size, the core-shell islands exhibit up to 40% higher $T_{b}$. These samples represent model systems for next generation high density storage magnetic media (HDSMM) since they consist of equally spaced magnetic nanodots with negligible mutual interactions and very uniform magnetic properties, such as magnetic moment, easy axis, and switching fields. Their density is with 15 Tera dots/in^2^ one order of magnitude higher than the one of present recording media.

## 2. Materials and Methods

The measurements were performed in an ultra-high vacuum (UHV) chamber equipped with a variable-temperature STM and an in-situ MOKE [25]. The Pt(111) surface was cleaned in vacuum by repeated cycles of Ar-ion sputtering (1.2 kV, 300 K, 3 μA/cm^2^), annealing in oxygen ($3\times 10^{-7}$ mbar, 800 K, 10 min), and flash annealing to 1300 K. The Au(11,12,12) surface was cleaned in vacuum by repeated cycles of Ar-ion sputtering (0.9 kV, 300 K, 2 μA/cm^2^) and annealing to 850 K. Co, Fe, Ag, Rh, and Pd were deposited by *e*-beam evaporation from high purity rods (99.995%) with fluxes optimized for each element, namely 0.13 mono-layers (ML)/min (Co), 0.15 ML/min (Fe), $3\times 10^{-4}$ ML/min (Ag), $9\times 10^{-4}$ ML/min (Rh), and 0.02 ML/min (Rh) (the error bar is 5–10% for each flux). The background pressure during sample preparation was below $8\times 10^{-11}$ mbar.

We used kinetically-controlled growth in order to define the island density, size, and shape [9]. The Co core islands are grown in two steps. The first step is performed at low deposition temperature $T_{\mathrm{dep}}$ determining the island density at the given deposition flux. The second deposition temperature is chosen higher (i) in order to have all atoms that land on top of the islands descend before they can create second layer nuclei, and (ii) to make sure that all atoms landing between the islands reach them and attach to them, instead of creating new ones. Lateral decoration with the elements Ag, Rh, and Fe is performed at deposition temperatures guaranteeing similar conditions as the second growth step of the Co core islands, i.e., atoms landing on top of Co core islands can descend, and atoms landing between islands must reach them and not create new nuclei. This way a perfect lateral decoration can be achieved in many cases, the only exception being Ag, where some degree of second layer nucleation could not be avoided. Capping with a perfect Pd mono-layer is achieved by creating many nuclei at low deposition temperature, followed by annealing.

The temperature dependence of the magnetic susceptibility has been measured with MOKE in polar geometry by applying an oscillatory out-of-plane field with 100 Oe amplitude and 31 Hz frequency. Lock-in detection has been used to measure the real and imaginary parts of the zero-field susceptibility, $\chi^{\prime }\left(T\right)$ and $\chi^{\prime \prime }\left(T\right)$, respectively. In all χ(T) figures, the $\chi^{\prime }\left(T\right)$-peak has been normalized to 1.

## 3. Results and Discussion

### 3.1. MAE in 3d–4d Two-Dimensional Islands

**Figure 1:**
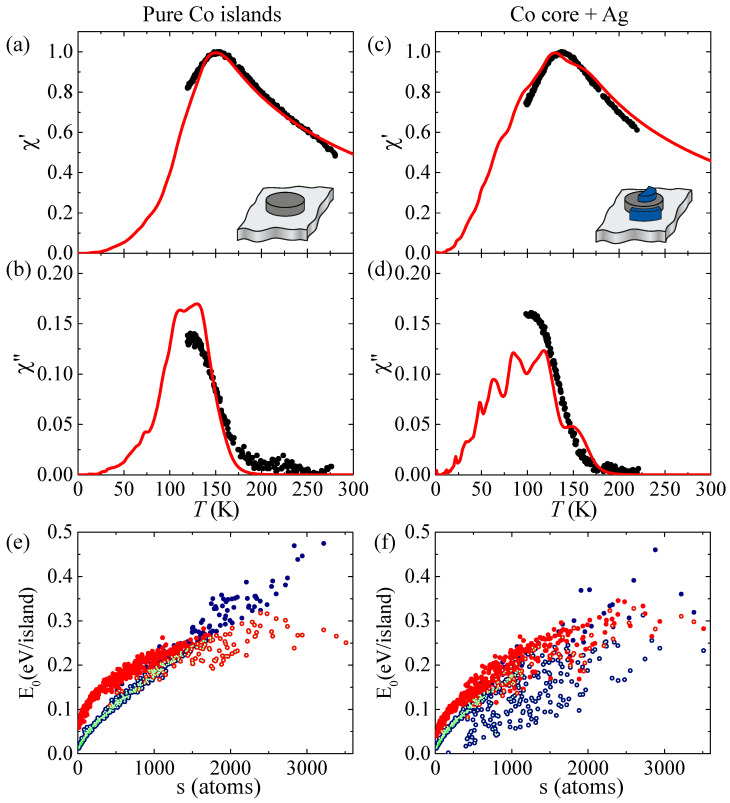
(**a**–**d**) Experimental and simulated out-of-plane zero-field susceptibility curves. (**a**,**b**) pure Co islands grown on Pt(111) in two steps, 0.10 ML at $T_{\mathrm{dep}}=130$ K and 0.15 ML at $T_{\mathrm{dep}}=280$ K. (**c**,**d**) The Co islands in (**a**,**b**) after partial decoration with Ag, 0.10 ML at $T_{\mathrm{dep}}=220$ K. Black dots: experiment; red lines: best fit of all four χ(T) curves with one set of parameters ($K_{s,\mathrm{Co}}=0.065$ meV/atom, $K_{p,\mathrm{Co}}=0.95$ meV/atom, $K_{s,\mathrm{Ag}}=0.16$ meV/atom, $K_{p,\mathrm{Ag}}=-0.12$ meV/atom). (**e**,**f**) Zero-field magnetization reversal energy for (**e**) pure Co islands and (**f**) Co islands partially decorated and capped with Ag. Blue dots represent the values for coherent rotation, $E_{\mathrm{CR}}$, and red for reversal by domain walls, $E_{\mathrm{DW}}$. Light green dots: lowest values selected for each island, $E_{0}$.

Figure 1a–d shows the temperature dependence of the real ($\chi^{\prime }\left(T\right)$) and imaginary ($\chi^{\prime \prime }\left(T\right)$) part of the magnetic susceptibility measured on pure Co islands and on Co-core Ag-decorated ones. The blocking temperature $T_{b}$ is defined as the maximum of $\chi^{\prime \prime }\left(T\right)$. A first qualitative inspection shows that $T_{b}$ of the ensemble of decorated islands slightly decreases, suggesting a reduction of the out-of-plane anisotropy. Relating these changes in $T_{b}$ to the MAE of the constituent atoms requires knowledge of the reversal mechanism and of the atomic scale morphology of the ensemble.

Magnetic nanostructures can reverse their magnetization either by coherent rotation (CR), where the magnetic moments of all constituent atoms stay aligned during the entire reversal process [27,28], or by nucleation and propagation of a domain wall (DW) [29,30,31], or by more complicated processes [32,33,34]. Fitting the experimental χ(T) data with the different models helps to elucidate the magnetization reversal mechanism. In this study, two magnetization reversal models have been considered for the simulations of the susceptibility, namely CR and DW; as we will see below, the island size and shape define which of the two takes place for a given island [35,36,37].

**Figure 2:**
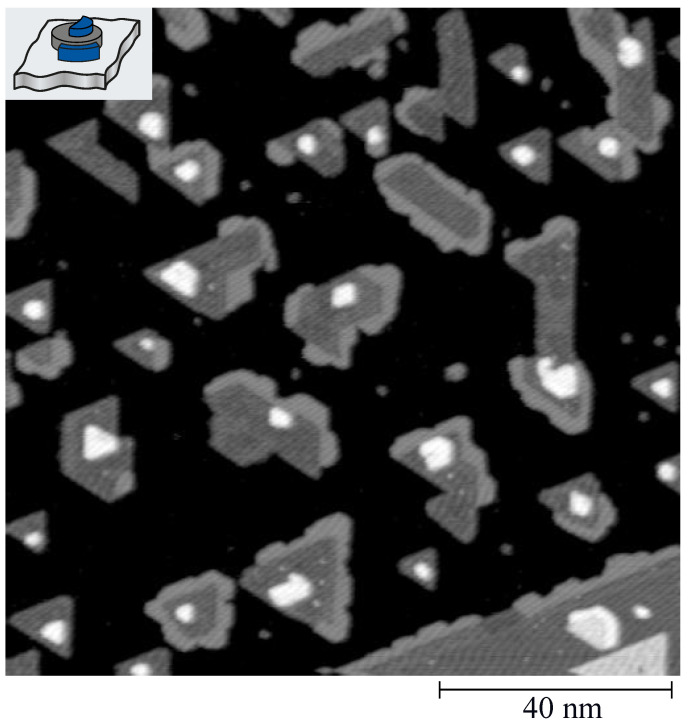
STM image of Co-core Ag-decorated islands on Pt(111). Co is deposited in two steps, 0.10 ML at $T_{\mathrm{dep}}=130$ K to define the island density with the addition of 0.15 ML at $T_{\mathrm{dep}}=280$ K to grow the existing nuclei to the desired size. Ag decoration is performed by depositing 0.10 ML Ag at $T_{\mathrm{dep}}=220$ K. Dark gray: Co islands, light gray: Ag decoration, white: Ag overlayer (tunnel voltage $V_{t}=-0.3$ V, tunnel current $I_{t}=1$ nA, T=300 K).

From several STM images, acquired at random places on the crystal surface, we extracted the island size *s*, perimeter *p*, and cross-section $W_{z}$ (all given in atoms) for an ensemble of at least 500 islands for each sample. For decorated islands, the perimeter is split in free $p_{f}$ and decorated $p_{d}$ parts; similarly, the island surface is split into free $s_{f}$ and capped $s_{c}$ surface areas. An example of decorated islands is shown in Figure 2 for the case of Ag decoration. Ag forms a non-uniform partial rim around the island edges with an apparent height of Δz=2.75±0.15 Å, which can easily be discerned from the one of Co of Δz=2.20±0.10 Å. This chemical contrast is clearly visible as brighter gray scale of the Ag rim in the top-view representation of the STM image. A similar chemical contrast is also observed for the other 4*d* elements, allowing us for all element combinations used to determine the numbers $p_{f}$, $p_{d}$, $s_{f}$, $s_{c}$, and $W_{z}$, quantifying the atomic scale morphology.

Since the islands in the ensemble have an irregular shape, $W_{z}$ was considered as the largest width in each island. For the CR model, the magnetization reversal energy is $E_{\mathrm{CR}}=sK_{\mathrm{eff},\mathrm{CR}}$, where $K_{\mathrm{eff},\mathrm{CR}}=\left[\left(s-p\right)K_{s}+pK_{p}\right]/s$ is the effective anisotropy with $K_{s}$, and $K_{p}$ the anisotropy energies per atom for interface and interline, respectively. To take partial island decoration (capping) into account, we define $K_{p}=\left(p_{f}K_{p,\mathrm{Co}}+p_{d}K_{p,4d}\right)/p$, and the same for the effective interface energy. For the DW model, we have: $E_{\mathrm{DW}}=4W_{z}\sqrt{A_{\mathrm{Co}-\mathrm{Co}}K_{\mathrm{eff},\mathrm{DW}}+A_{\mathrm{Co}-4d}K_{\mathrm{eff},\mathrm{DW}}}$, where $K_{\mathrm{eff},\mathrm{DW}}=\left[\left(W_{z}-2\right)K_{s}+2K_{p}\right]/W_{z}$, and $A_{\mathrm{Co}-\mathrm{Co}}$ and $A_{\mathrm{Co}-4d}$ are the Co-Co and Co-4*d* exchange stiffnesses. Additional parameters are the lattice parameter for the atoms in an island, taken identical to the Pt(111) one $d_{\mathrm{nn}}=2.775$ Å since the islands grow pseudomorphic with the substrate, the reduced exchange stiffness between Co atoms due to the lower dimensionality of the atoms in the islands $A_{\mathrm{Co}-\mathrm{Co}}=15$ pJ/m [35,38], and the pre-exponential factor in the Boltzmann term for the magnetization reversal frequency that we set to $\nu_{0}=5\times 10^{11}$ Hz.

We calculate the zero-field magnetization reversal energies $E_{\mathrm{CR}}$ and $E_{\mathrm{DW}}$ for both reversal mechanisms and the exact morphology of each individual island for a specific set of $K_{s}$ and $K_{p}$ parameters. This results in the $E_{0}$ distributions shown in Figure 1e,f for pure Co and Ag-decorated Co-core islands. Each dot is one island. For each island, we retain the smallest energy between the two reversal modes, and these dots are marked in green. The contribution of each island is then summed up, producing $\chi^{\prime }\left(T\right)$ and $\chi^{\prime \prime }\left(T\right)$ curves, as described in our previous work [35]. The evaluation of the agreement between experiment and simulation was performed by visual inspection. We focused primarily on the correct reproduction of the $T_{b}$ peak position and, secondly, on the shape of the curves. The error bars in the $K_{s}$ and $K_{p}$ were derived by investigating changes in the χ(T) curves from small variations around the optimum value.

The lack of atomic resolution in the STM topographies induces a larger uncertainty in the island perimeter evaluation compared to the surface. Kink sites, corners, and, in general, the exact location of the perimeter atoms remain hidden; thus, the measured perimeter is a mean estimation of the real perimeter length. The exact atomic structure of the perimeter strongly depends on the growth parameters, such as temperature and flux. In addition, atoms at the perimeter with different coordination are expected to contribute differently to the island MAE [7]. This implies that islands having the same perimeter and area in the STM images but grown with different parameters can show slightly different magnetic properties. This uncertainty on the island morphology is reflected on the *K* parameters, which can slightly vary from one sample to the other. For this reason, $K_{p,\mathrm{Co}}$ and $K_{s,\mathrm{Co}}$ have to be evaluated by fitting the χ(T) curves for each sample. In addition, this uncertainty in the perimeter structure affects the value of $K_{p}$ of the decorating element. To reduce this uncertainty, we simultaneously fit the χ(T) curves of pure Co islands and decorated islands in order to obtain the best estimation for the four *K* values, $K_{p,\mathrm{Co}}$, $K_{s,\mathrm{Co}}$ and $K_{p,4d}$, $K_{s,4d}$.

Between different epitaxial growth experiments, variations of the order of 5 to 10% in the deposition flux are observed. This resulting imprecision in the surface coverage causes variations of $T_{b}$ for nominally identical samples. For example, for pure Co islands with $\Theta_{\mathrm{Co},\mathrm{total}}=0.25$ ML, $T_{b}$ varies in the range 115±15 K. Therefore, the χ(T) curves for pure Co islands were always acquired in every experiment as reference in order to observe the exact change in $T_{b}$ after decoration or capping with a given element.

Looking at the zero-field magnetization reversal energies $E_{\mathrm{CR}}$ and $E_{\mathrm{DW}}$ in Figure 1e,f, one sees that, for pure Co islands, there is a clear transition from CR to DW at about 1500 atoms size, whereas the Ag decorated islands reverse by CR for all sizes. The simulated $\chi^{\prime }\left(T\right)$ and $\chi^{\prime \prime }\left(T\right)$ curves agree very well with the measured ones for pure and Ag decorated Co islands. For pure Co islands, we find $K_{s,\mathrm{Co}}=0.065\pm 0.010$ meV/atom and $K_{p,\mathrm{Co}}=0.95\pm 0.05$ meV/atom, in agreement with our previous works [8,26]. For the Co core of the Ag decorated islands, we take this $K_{s,\mathrm{Co}}$ value and find that the Co/Ag interface contributes positively to the MAE with $K_{s,\mathrm{Ag}}=0.16\pm 0.02$ meV/atom, in agreement with previous studies [19,21,22], while the Co/Ag interline produces a negative MAE of $K_{p,\mathrm{Ag}}=-0.12\pm 0.03$ meV/atom. Due to the low spin polarizability of Ag, we fixed the exchange stiffness $A_{\mathrm{Co}-\mathrm{Ag}}$ to zero in these simulations.

**Figure 3:**
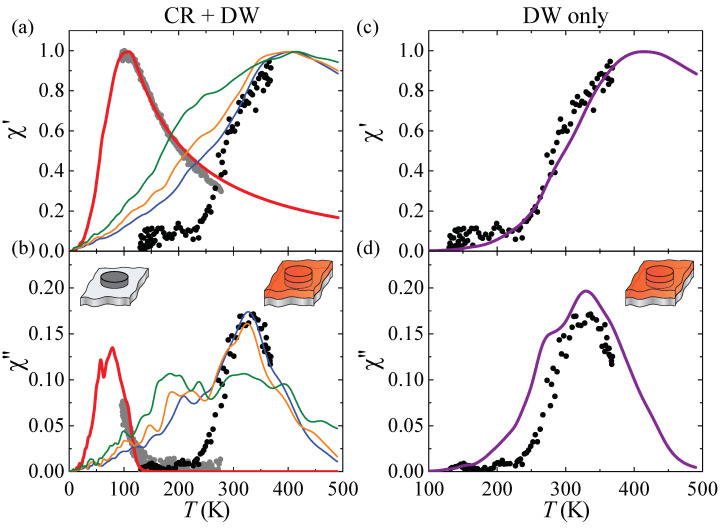
Out-of-plane zero-field susceptibility curves for pure Co islands (gray) and fully Pd capped ones (black) on Pt(111) ($\Theta_{\mathrm{Co}}=0.10$ ML at $T_{\mathrm{dep}}=130$ K, $\Theta_{\mathrm{Co}}=0.08$ ML at $T_{\mathrm{dep}}=280$ K, capping with 1.0 ML Pd at $T_{\mathrm{dep}}=90$ K, annealing at $T_{\mathrm{ann}}=350$ K). Full curves are simulations using (**a**,**b**) CR and DW models, and (**c**,**d**) DW only. (**a**,**b**) Thick red lines: simulation for pure Co islands ($K_{s,\mathrm{Co}}=0.065$ meV/atom, $K_{p,\mathrm{Co}}=0.95$ meV/atom). Blue lines: $K_{s,\mathrm{Pd}}=1.0$ meV/atom, $A_{\mathrm{Co}-\mathrm{Pd}}=0.75$ pJ/m; orange lines: $K_{s,\mathrm{Pd}}=0.9$ meV/atom, $A_{\mathrm{Co}-\mathrm{Pd}}=1.5$ pJ/m, green lines: $K_{s,\mathrm{Pd}}=0.8$ meV/atom, $A_{\mathrm{Co}-\mathrm{Pd}}=1.5$ pJ/m. (**c**,**d**) Purple lines: $K_{s,\mathrm{Pd}}=0.75$ meV/atom, $A_{\mathrm{Co}-\mathrm{Pd}}=3$ pJ/m. In all cases, $A_{\mathrm{Co}-\mathrm{Co}}=15$ pJ/m, $K_{p,\mathrm{Pd}}=0.5$ meV/atom.

As can be seen upon inspection of Figure 3a,b, the magnetization reversal kinetics of pure Co islands is again perfectly reproduced in the simulations, and this with the same atomic parameters $K_{s,\mathrm{Co}}$ and $K_{p,\mathrm{Co}}$ as above, showing the consistency of our approach. Note that the pure Co islands are now smaller than above and, therefore, have lower $T_{b}$. Pd capping shifts $T_{b}$ to above room temperature. Therefore, the interface between Pd and Co must have a significant out-of-plane MAE. The fits using a transition from CR to DW fit $\chi^{\prime \prime }$ very well but fail to fit the initial increase in $\chi^{\prime }$. A fit with DW only perfectly reproduces $\chi^{\prime }$ and is in good agreement with the overall shape and position of $\chi^{\prime \prime }$. This fit yields $K_{s,\mathrm{Pd}}=0.75$ meV/atom for the Co/Pd interface, roughly twice the value reported for Co/Pd thin film multi-layers [39,40], $K_{p,\mathrm{Pd}}=0.50$ meV/atom for the Co/Pd interline, i.e., a reduction by −0.45 meV/atom compared to Co step atoms, and an exchange stiffness $A_{\mathrm{Co}-\mathrm{Pd}}=3$ pJ/m.

Data on the Co/Rh interface (not shown) reveal $K_{s,\mathrm{Rh}}=0.08$ meV/atom, almost an order of magnitude larger than previously reported [41] and of reversed sign with respect to the Co/Rh(111) case [11], $A_{\mathrm{Co}-\mathrm{Rh}}=1.5$ pJ/m and $K_{p,\mathrm{Rh}}=-0.3$ meV/atom. Finally, the dependence of $T_{b}$ with the number of Pd capping layers reveals that the maximum MAE is achieved at about 2 mono-layers of Pd, in agreement with previous studies on thin films [42,43].

**Figure 4:**
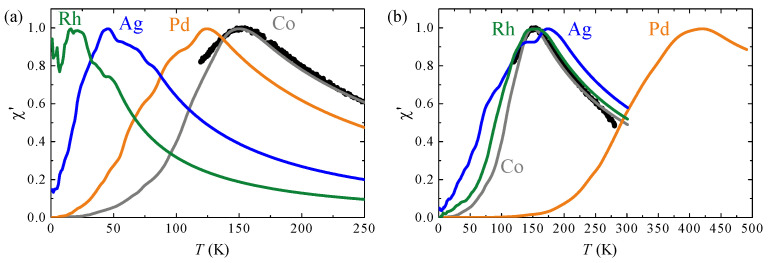
Predicted $\chi^{\prime }\left(T\right)$ curves for 0.25 ML Co islands on Pt(111) perfectly (**a**) decorated and (**b**) capped with the studied 4*d* elements. The curves have been calculated using the respective atomic anisotropy energies derived above. Black symbols: experimentally measured $\chi^{\prime }\left(T\right)$ for islands corresponding to 0.25 ML Co.

Our findings on the effect of the bimetallic interfaces and interlines investigated for islands grown on Pt(111) are summarized in Figure 4, showing the calculated $\chi^{\prime }\left(T\right)$ curves for an ensemble of Co islands perfectly laterally decorated (Figure 4a) or completely capped (Figure 4b). Rh, Ag, and Pd interlines with Co weaken the MAE compared to Co step atoms facing vacuum, whereas Ag capping weakly and Pd capping strongly enhance the MAE, bringing $T_{b}$ well beyond room temperature for islands containing only 1000 Co atoms.

### 3.2. CoFe Nanoislands on Au(11,12,12)

A model system for next generation HDSMM requires an ordered array of magnetic nanodots having negligible mutual interactions and uniform magnetic properties, such as magnetic moment, easy axis, and switching field. It has been demonstrated that Au(111) vicinal surfaces can be used to self-assemble highly ordered arrays of close to monodisperse Co nanoislands with densities of 26 or 15 Tdots/in^2^ for Au(7,8,8) or Au(11,12,12), respectively, fulfilling all these requirements [23,24,25]. The main drawback is the relatively low achieved $T_{b}$ compared to room temperature. The islands in these superlattices had only a few hundred atoms size; therefore, they reverse their magnetization by CR. For this reversal mechanism, the magnetization reversal energy coincides with the MAE that is proportional to $T_{b}$ [8]. $T_{b}$ is around 45 K in islands composed of 245 Co atoms, and it increases to 85 K for islands of 600 Co atoms [24,25]. One possibility to enhance $T_{b}$, keeping the size constant, consists is using bimetallic islands combining 3*d* elements, with high spin, and 4*d* or 5*d* materials [44,45] having high spin-orbit coupling. However, 4*d* and 5*d* elements are usually characterized by low magnetic moments. Small magnetic moments per island, however, result in undesirably high switching fields ($H_{s}=2K/M$), which can be larger than the technically achievable writing fields. However, previous studies have shown that also a 3*d*–3*d* combination may raise the MAE; for 1 ML thin CoFe alloy films on Pt(111), the MAE is strongly enhanced for 50% alloy composition due to the electronic hybridization of the Fe and Co *d*-orbitals [46]. 3*d* elements are characterized by large magnetic moments, thus resulting in a large *M* value; therefore, one combines thermal stability (high MAE) with accessible switching fields.

**Figure 5:**
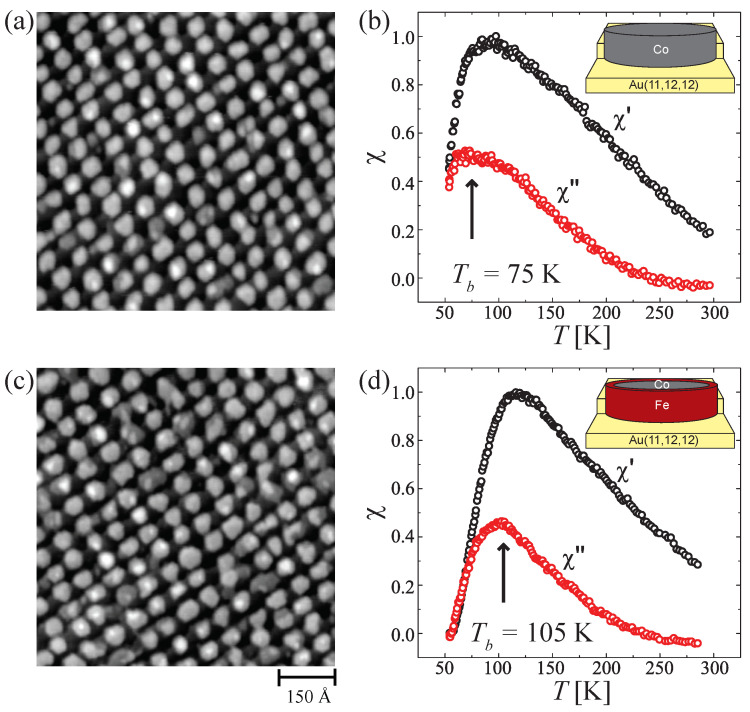
Morphology and magnetism of pure Co and Co-core Fe shell nanoislands self-assembled on Au(11,12,12). (**a**) STM and (**b**) MOKE measurements of pure Co islands ($\Theta_{\mathrm{Co}}=0.9$ ML) and (**c**,**d**) of Co-core Fe-shell islands ($\Theta_{\mathrm{Co}}=0.6$ ML, $\Theta_{\mathrm{Fe}}=0.3$ ML). Insets: island composition sketches. Adapted from ref. [48].

**Figure 6:**
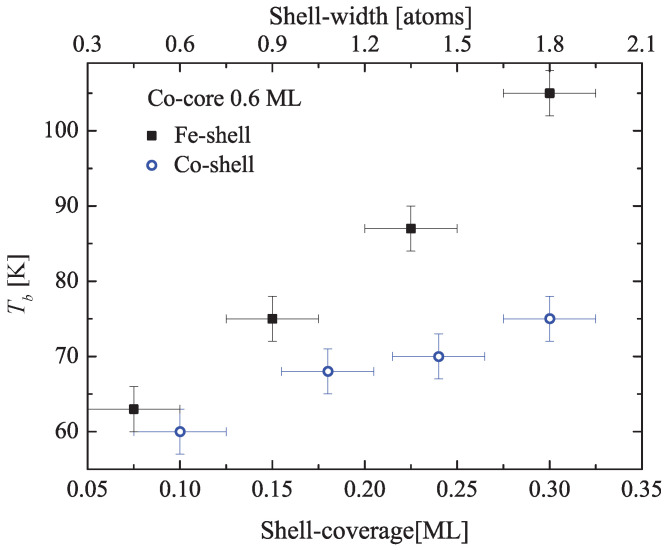
Blocking temperature ($T_{b}$) of Co-core Fe-shell islands (black filled squares) and of pure Co islands (blue open circles) as a function of the shell width. Top axis: shell-width in atoms calculated assuming a homogeneous decoration of the Co-core.

Figure 5 compares morphology and magnetism of pure Co (Figure 5a,b) and Co-core Fe-shell (Figure 5c,d) islands grown on Au(11,12,12). Both island types are two atomic layers thick and have identical size and, therefore, vary only in composition. The optimum spatial order is obtained when Co is deposited in steps of 0.15 ML at 130 K with subsequent annealing to 400 K up to the desired coverage. For Fe, the deposition temperature was 200 K and the annealing temperature was limited to 300 K in order to avoid alloying. The maximum coverage has been fixed to 0.9 ML in order to remain below the coalescence threshold that in the case of pure Co islands is around 1.1 ML [25], while this threshold is slightly reduced for Fe [47]. Following this procedure, the two morphologies are hard to distinguish; in both cases, they consist of well separated bi-layer dots composed, on average, of 490±80 atoms (Figure 5a,c) as concluded from the island area assuming pseudomorphic growth. On the contrary, $T_{b}$ is strongly different in the two systems; it increases from 75 K for pure Co islands to 105 K for the bimetallic islands, which represents a 40% enhancement of the energy barrier separating the two magnetization orientations up and down. The $T_{b}$ dependence on the shell thickness is shown in Figure 6, for a fixed Co-core size of 330 atoms (0.6 ML). $T_{b}$ rises linearly with a slope steeper than the one observed for a Co-shell (pure Co-islands) used as a reference.

This behavior excludes a pure size effect, since islands with the same number of atoms but different chemical composition have markedly different magnetization reversal kinetics. We attribute the increase of $T_{b}$ generated by the Fe shell to electronic hybridization between *d*-orbitals of Co and Fe taking place at the core-shell interline. Electronic structure calculations for CoFe mono-layers on Pt(111) revealed that the increase of MAE observed at 50% composition is due to the fine tuning of the occupation numbers of spin-down $d_{x^{2}-y^{2}}$ and $d_{xy}$ in-plane orbitals, both for Fe and Co, while the occupation of the $d_{3z^{2}-r^{2}}$, $d_{xz}$, and $d_{yz}$ out-of-plane orbitals remains nearly unchanged upon change of the composition [46]. Very likely, a similar electronic hybridization takes place at the Fe-Co interline in our bimetallic islands. Different from the previous section on 3*d*–4*d* islands, here, we do not see a clear chemical contrast between Co and Fe; consequently, we refer to the MAE of the entire island simply as *K*, without distinguishing the atomic interline and interface contributions.

Au decoration of Co bi-layer islands on Au(111) was reported to enhance *K* by 5–11% [49], which was attributed to strain inside the Co islands caused by the Au shell. While we can not exclude the contribution of such effects in our sample, they cannot be the main mechanism of our much larger enhancement of *K* by 40%. We note that Au atoms interfacing Co can have large induced moments [50]. Therefore, the effect reported in reference [49] may also entirely be explained by the spin-orbit coupling of the induced Au moment increasing *K* and, thereby, $T_{b}$.

**Figure 7:**
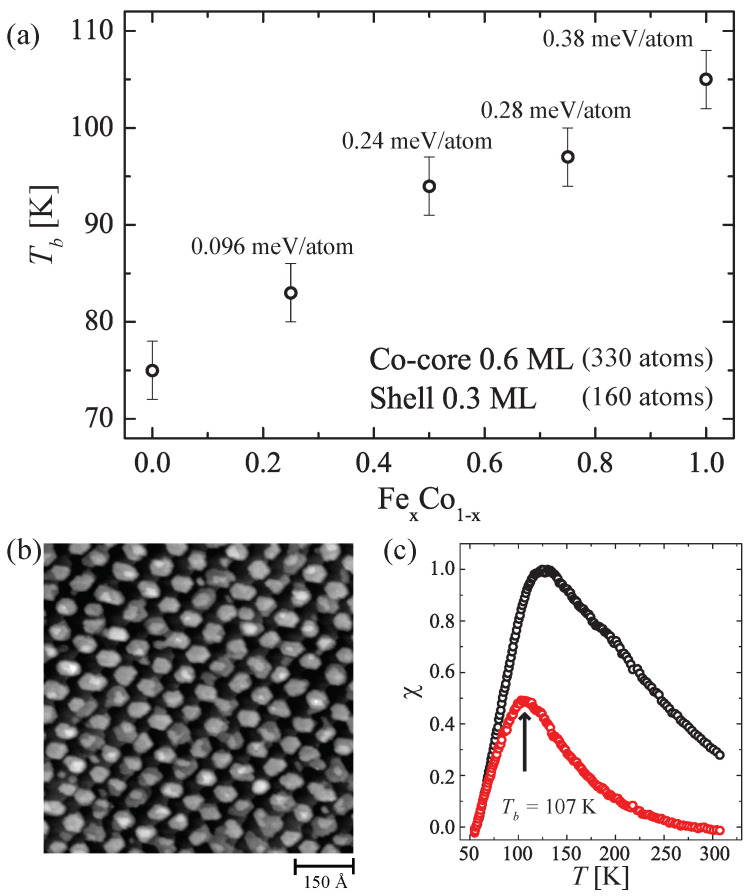
Co-core Fe_x_Co_1−x_-shell islands on Au(11,12,12). (**a**) $T_{b}$ as a function of the shell composition *x*. The labels give the enhancement of the MAE due to the Fe fraction *x* in the shell. (**b**) STM image and (**c**) $\chi^{\prime }\left(T\right)$ (black) and $\chi^{\prime \prime }\left(T\right)$ (red) of 0.45 ML Co-core 0.45 ML Fe_0.5_Co_0.5_-shell islands.

Now, we turn to islands with alloy shells around the Co-cores. Figure 7a reports the dependence of $T_{b}$ on the alloy stoichiometry for Co-core Fe_x_Co_1−x_-shell islands on Au(11,12,12). The island core contains the usual amount of 0.6 ML Co to allow for a direct comparison with the results of Figure 6. The shell was grown by co-depositing Co and Fe, and the stoichiometry was obtained by adjusting the relative deposition fluxes. This procedure was repeated 3 times in coverage steps of 0.1 ML at 180 K and subsequent annealing at 350 K.

Clearly, increasing the Fe content in the shell increases $T_{b}$, with the highest value found for the pure Fe shell. Since the islands reverse by CR [25,34], we can convert the $T_{b}$ variation into *K* variations due to the core-shell interline or to the alloy. The rate of barrier crossing for an ensemble of monodisperse nanoislands with the same magnetization reversal energy *K* and magnetization *M* in an external magnetic field *H* is given by $\nu =\nu_{0}\exp\left(\left(-K\pm HM\right)/k_{B}T\right))$ [8], the attempt frequency is set as above to $\nu_{0}=5\times 10^{11}$ Hz, $k_{B}$ is the Boltzmann constant, and *T* is the temperature. In the limit of zero-field measurements, one obtains $\Delta K=k_{B}\Delta T_{b}\;ln\left(\nu_{0}/\nu_{\mathrm{mes}}\right)$, where $\nu_{\mathrm{mes}}=31$ Hz is the field modulation frequency used in MOKE. The shell width is about two atoms wide, meaning that the inner atom of the shell is facing the Co core, while the outer one is facing vacuum. This implies that their electronic and magnetic properties are different. However, for simplicity and because our measurements do not allow to discriminate between the two, we assume that all atoms in the shell give the same contribution to ΔK. This contribution is shown in Figure 7a as a function of the shell stoichiometry. Co-Fe interlines are found to be more efficient, by a factor of 1.5 in increasing *K*, than a random Fe_50_Co_50_ alloy.

Bigger shells would be necessary to further increase $T_{b}$. Unfortunately, due to the worse self-assembly of Fe [47], it is very difficult to prepare samples with high Fe content in the magnetic nanostructures without creating their coalescence. We achieved with $T_{b}$ of 107 K the highest blocking temperature in 0.45 ML Co-core and 0.45 ML Fe_0.5_Co_0.5_-shell islands, corresponding to an equal size of 245 atoms for both core and shell. Figure 7b,c show an STM image and their zero-field susceptibility. The relative change in the energy barrier is 0.25 meV/shell atom, in very good agreement with what was found for the 0.6 ML Co-core case. If it were possible to decorate 0.45 ML Co-core islands with a pure 0.45 ML Fe shell without coalescence, the expected blocking temperature would be even higher, and extrapolating the results of Figure 7a would yield a theoretical value of $T_{b}=120$ K.

In order to compare the performance of the aforementioned 2D model systems with real magnetic recording media and other model systems, it is useful to consider the alignment of easy magnetization axes, the magnetic anisotropy *K* per atom, and the magnetic moment *M* per atom, with K/M defining the switching field. For recording media operating at room temperature, the requested data retention times can be guaranteed with K=1.2 eV/grain. How many grains are needed for a bit depends on the uniformity of the easy axes. A single grain suffices for uniaxial and sufficiently monodisperse systems. For 3D colloid nanoparticles, such as FePt in the L1_0_ phase (4 nm diameter) [51], *K* per atom is very high, but they are not uniaxial, bearing problems with dipolar magnetic interactions, and, for bit uniformity of the magnetization, many nanoparticles per bit are required. The samples discussed in the present paper are uniaxial and sufficiently monodisperse for single magnetic dot bits; however, their *K* is too small for the given size by a factor of 3–5, therefore they would require storage operation at 100 K. The fact that *K* is small in our nanostructures is due to them being 2D; therefore, at densities improving the ones of present media, they cannot host as many atoms as 3D particles. Growing them into the third dimension, e.g., by piling them up in successive growth steps [52], one can easily achieve the required *K*; however, the growth methods to do this without losing order remain yet to be discovered.

## 4. Conclusions

We studied, for bimetallic two-dimensional islands grown on single crystal surfaces, how the hybridization between different elements at the island edge (interline) and surface (interface) modifies the magnetic anisotropy energy, and, consequently, the thermal magnetization reversal. We focused on Co-core islands decorated by 3*d* (Fe) and 4*d* (Ag, Rh, and Pd) transition metals. These islands were grown on Pt(111), where they were randomly spaced and exhibited a broad size distribution, and on vicinal Au(111) surfaces, that act as templates for the nucleation of Co, giving rise to equally spaced and almost mono-dispersed islands. By combining morphological characterization of the island ensemble by means of STM with temperature-dependent magnetic susceptibility measurements performed with MOKE on the very same samples, we could extract the interline and interface anisotropies originating from Co atoms interfaced with the different elements. We used this information to realize ultra-dense island superlattices (15 Tdots/in^2^), consisting of only a few hundred atoms and, nevertheless, displaying blocking temperatures above 100 K.

Fundamental studies, as the one presented here, enable predictions on which structures to aim for in future magnetic recording materials. We took great care that the interlines and interfaces are abrupt in avoiding growth or annealing temperatures where inter-diffusion of the elements takes place. Only in one example did we create alloy shells to see that their anisotropy is lower than the one of interlines, suggesting that onion-type magnetic nanostructures with a periodic alternation of two elements have higher blocking temperatures than homogenous alloys of the same elements. We emphasize that the interline and interface anisotropies refer to the (111) direction of the growth substrate, and the same elements are expected to exhibit different such anisotropies for other surface orientations. We also emphasize that vertical and lateral inter-diffusion have very different effects on the magnetic properties, opening up alternative ways to tailor the MAE of magnetic nanostructures.

The largest anisotropies per atom reported here are for Co step atoms and for Co atoms interfacing Pt(111) below and Pd on top. The values are of the order of K=1 meV per atom, implying that the smallest single domain grains fulfilling the thermal stability criterion of magnetic recording materials would have to contain 1200 atoms. Much larger magnetic anisotropies per atom can be realized for low-coordinated atoms. For example, single atoms adsorbed on fcc(111) surfaces have only 3 neighbors below and exhibit anisotropy energies per atom of up to K=9 meV [7]. Even lower effective coordination can be obtained when the magnetic adatom binds mostly to a single atom of the substrate. This is the case for MgO(100) thin films grown on Ag(100), where the magnetic atoms bind predominantly to O and only very weakly to the 4 neighbor Mg atoms. Such systems, therefore, offer basically one-fold coordination, creating a close to uniaxial crystal field. The largest anisotropy per atom for a 3*d* element was obtained for Co with a value of K=57 meV [53], representing the theoretical limit of orbital moment times SOC. The largest anisotropy reported, presently, for a single surface adsorbed atom is K=250 meV and has been achieved tor the rare-earth element Dy, again adsorbed onto the one-fold coordinated O sites of MgO/Ag(100) [54]. However, these exceptionally high values of single atoms require the low coordination that establishes a large orbital magnetic moment that is at the origin of the large magnetic anisotropy. Every attempt to combine several of these atoms to achieve a stable ferromagnetically ordered bit increases their coordination and decreases their anisotropy. Unless ways of strong magnetic exchange coupling without increase of coordination are found, magnetic recording materials will have to be composed of atoms that have a magnetic anisotropy energy of around 1 meV.

## Data Availability

Data are contained within the present article.
